# Supplementary material for: Smooth Interpolating Curves with Local Control and Monotone Alternating Curvature
Source: Comput Graph Forum. 2022 Oct 6;41(5):25–38. doi: 10.1111/cgf.14600 (PMC9827861; doi:10.1111/cgf.14600)
Supplement: Supplementary file 1 — Supplement Material [file CGF-41-25-s001.zip › Local-Smooth-Interpolating-MonoCurvature/extern/clothoids/submodules/Utils/docs/api-cpp/program_listing_file_Utils_Token.hxx.html]

Program Listing for File Token.hxx — Utils v1.2

### Navigation

- index
- toc
- Utils »
- Program Listing for File Token.hxx

# Program Listing for File Token.hxx¶

↰ Return to documentation for file (`Utils/Token.hxx`)

```
/*--------------------------------------------------------------------------*\
 |                                                                          |
 |  Copyright (C) 2017                                                      |
 |                                                                          |
 |         , __                 , __                                        |
 |        /|/  \               /|/  \                                       |
 |         | __/ _   ,_         | __/ _   ,_                                |
 |         |   \|/  /  |  |   | |   \|/  /  |  |   |                        |
 |         |(__/|__/   |_/ \_/|/|(__/|__/   |_/ \_/|/                       |
 |                           /|                   /|                        |
 |                           \|                   \|                        |
 |                                                                          |
 |      Enrico Bertolazzi                                                   |
 |      Dipartimento di Ingegneria Industriale                              |
 |      Universita` degli Studi di Trento                                   |
 |      email: enrico.bertolazzi@unitn.it                                   |
 |                                                                          |
\*--------------------------------------------------------------------------*/


#pragma once

#ifndef DOXYGEN_SHOULD_SKIP_THIS
#ifndef UTILS_TOKEN_dot_HXX
#define UTILS_TOKEN_dot_HXX
#endif

//
// https://stackoverflow.com/questions/53849/how-do-i-tokenize-a-string-in-c
//

namespace Utils {

  class Tokenizer {
  protected:
    size_t       m_offset;
    string const m_string;
    string       m_token;
    string const m_delimiters;
  public:
    Tokenizer( string const & str, string const & delimiters )
    : m_offset(0)
    , m_string(str)
    , m_delimiters(delimiters)
    { }

    string get_token() const { return m_token; }

    bool next_token();
  };

  void
  split_string(
    string const   & str,
    string const   & sep,
    vector<string> & arr
  );

}


#endif
```

### Quick search

### Table of Contents

- rang - Colors for your Terminal
- Zstr
- C++ API

«
hide menu

menu
sidebar
»

### Navigation

- index
- toc
- Utils »
- Program Listing for File Token.hxx

© Copyright 2021, Enrico Bertolazzi.
Created using Sphinx 4.2.0.
